# Seasonal variations of insulin sensitivity from a euglycemic insulin clamp in elderly men

**DOI:** 10.3109/03009734.2011.628422

**Published:** 2012-02-15

**Authors:** Lars Berglund, Christian Berne, Kurt Svärdsudd, Hans Garmo, Håkan Melhus, Björn Zethelius

**Affiliations:** ^1^Department of Public Health/Geriatrics, Uppsala University, Uppsala, Sweden; ^2^Department of Medical Sciences, Uppsala University, Uppsala, Sweden; ^3^Department of Public Health/Family Medicine and Clinical Epidemiology Uppsala University, Uppsala, Sweden; ^4^Uppsala Clinical Research Center, University Hospital, Uppsala, Sweden; ^5^Medical Product Agency, Uppsala, Sweden

**Keywords:** Insulin secretion, insulin sensitivity, seasonal variation

## Abstract

**Introduction.:**

Seasonal variations in hemoglobin-A1c have been reported in diabetic patients, but the underlying mechanisms have not been elucidated.

**Aims.:**

To study if insulin sensitivity, insulin secretion, and fasting plasma glucose showed seasonal variations in a Swedish population-based cohort of elderly men.

**Methods.:**

Altogether 1117 men were investigated with a euglycemic insulin clamp and measurements of fasting plasma glucose and insulin secretion after an oral glucose tolerance test. Values were analyzed in linear regression models with an indicator variable for winter/summer season and outdoor temperature as predictors.

**Results.:**

During winter, insulin sensitivity (M/I, unit = 100 × mg × min^-1^ × kg^-1^/(mU × L^-1^)) was 11.0% lower (4.84 versus 5.44, *P* = 0.0003), incremental area under the insulin curve was 16.4% higher (1167 versus 1003 mU/L, *P* = 0.007). Fasting plasma glucose was, however, not statistically significantly different (5.80 versus 5.71 mmol/L, *P* = 0.28) compared to the summer season. There was an association between outdoor temperature and M/I (0.57 units increase (95% CI 0.29–0.82, *P* < 0.0001) per 10°C increase of outdoor temperature) independent of winter/summer season. Adjustment for life-style factors, type 2 diabetes, and medication did not alter these results.

**Conclusions.:**

Insulin sensitivity showed seasonal variations with lower values during the winter and higher during the summer season. Inverse compensatory variations of insulin secretion resulted in only minor variations of fasting plasma glucose. Insulin sensitivity was associated with outdoor temperature. These phenomena should be further investigated in diabetic patients.

## Introduction

HbA1c has been reported to show seasonal variations in diabetic patients (1–3). Whether corresponding seasonal variations of insulin sensitivity occur is largely unknown. Studies are inconclusive, with some demonstrating higher insulin sensitivity during the warm season (4), whereas others (5) fail to find a seasonal variation. Bunout et al. (6) find decreased insulin sensitivity during the warm season in elderly people. These studies use repeated measures designs but have limitations in small numbers of participants and in using insulin sensitivity measurements by surrogate methods based on fasting serum insulin concentrations.

Exposure to variations of outdoor temperature (ODT), one possible determinant of seasonal variations of insulin sensitivity, is more pronounced in Sweden, located in the subarctic and the northern part of the temperate climate zone, as compared to countries located in the southern parts of the temperate and in the tropical climate zones. There is a 21°C difference between the average temperatures for the extreme months of July and February in the middle part of Sweden (http://www.smhi.se). Basal metabolic rates co-vary with ODT (7), and heat treatment of rats increases skeletal muscle insulin sensitivity (8). Little is known of the effect of low ODT on human insulin sensitivity. A study under Swedish climatic conditions, with measurements of insulin sensitivity covering the whole calendar year, was a suitable strategy to investigate possible seasonal variations of insulin sensitivity.

Insulin sensitivity, insulin secretion, and their interaction are underlying mechanisms in type 2 diabetes and its development (9), with a particularly high type 2 diabetes risk for the combination of the lowest tertiles of insulin sensitivity and insulin secretion. For normal glucose tolerance individuals, insulin secretion and insulin sensitivity are linked through a negative feedback loop, whereby pancreatic beta cells compensate for changes in whole-body insulin sensitivity through a proportionate and reciprocal change in insulin secretion (10,11). Therefore, we sought to investigate if insulin sensitivity oscillations over the calendar year were mirrored by inverse insulin secretion variations in a population-based setting.

The aim of this study was to examine if seasonal variations (defined as winter/summer season) of fasting plasma glucose (FPG) and insulin-mediated glucose uptake using the gold standard euglycemic insulin clamp technique occurred in a population-based cohort of 71-year-old men and if corresponding variations of insulin secretion occurred, when measured by the incremental area under the insulin curve (AUC) during a 2-h oral glucose tolerance test (OGTT).

## Materials and methods

### Ethics statement

The study was approved by the local ethics committee, and all participants gave their informed consent. The Uppsala Longitudinal Study of Adult Men (ULSAM) was started in 1970 before any legislation on ethics approval was in place in Sweden. The study was performed in accordance with ethical guidelines at that time, including the Helsinki Declaration (WMA 1964), and a verbal consent documented in the patient file was considered to be a correct procedure. Later on, participants were invited for reinvestigations and at the third cycle (1991–1995), constituting the data included in this publication, a written informed consent was obtained as well as the approval of the local ethics committee at the Uppsala University, Faculty of Medicine. Further, approval of and informed consent to link national register data has also been obtained in writing. The full name of the ethics review board and its address at the time of approval: Local Ethics Committee, Faculty of Medicine, Uppsala University, Uppsala, Sweden. Today: Regionala etikprövningsnämnden in Uppsala, Box 1964, 751 49 Uppsala, Sweden. The change was made due to the Act (2003:460) concerning the Ethical Review Act. These changes came into force in 2004, implementing the EU-directive 2001/20 into Swedish law.

### Main study

ULSAM (http://www.pubcare.uu.se/ULSAM) is a population-based longitudinal study, still on-going for data collection and reinvestigations at present at age 88 years. All men born between 1920 and 1924 and resident in the municipality of Uppsala, located in the middle of Sweden, were invited to participate in a health survey at age 50 years. They were examined at the ages of 50 (*n* = 2322, participation rate 81.7%), 60 (*n* = 1860), 71 (*n* = 1221), 77 (*n* = 839), and 82 (*n* = 530) years. All examinations were made at the out-patient clinic for obesity and metabolic diseases at Uppsala University Hospital.

The present analysis included data from men examined at age 71 years between October 1991 and May 1995. Diabetic participants with insulin treatment were excluded. Plasma insulin and glucose measurements were from a 75-g OGTT and insulin sensitivity from a euglycemic insulin clamp investigation (*n* = 1117).

### Reliability study

At age 71 years, a subgroup of 20 participants was investigated twice within 4–6 weeks to determine intra-individual variations comprising the sum of technical measurement errors and biological variations.

### Anthropometric measurements

Height was measured to the nearest whole centimeter, and body weight to the nearest 0.1 kg. The body mass index (BMI) was calculated as the ratio of the weight (in kilograms) to the height (in meters squared). The waist circumference (WC) was measured midway between the lowest rib and the iliac crest.

### Data from questionnaires

A validated, optically readable, pre-coded, 7-day food record was completed by 1050 of the 1117 men for assessment of habitual dietary intake. Total energy intake (kilocalories) was calculated as the mean of the intakes over the 7 days (12).

Medication, smoking, and usual leisure-time physical activity were ascertained through self-report by questionnaires. High usual leisure-time physical activity (PA) was defined as engagement in any active recreational sports or heavy gardening at least 3 hours every week or regular engagement in hard physical training or competitive sport (13).

### Biochemical measurements

From an OGTT at age 71 years, blood samples were drawn immediately before (FPG) and 30, 60, 90, and 120 min after ingestion of 75 g anhydrous D-glucose dissolved in 300 mL water. Plasma insulin was assayed using an enzymatic immunological assay (Enzymmun, Boehringer Mannheim, Mannheim, Germany) gauged in an ES300 automatic analyzer (Boehringer Mannheim). Plasma glucose was measured by a glucose dehydrogenase method (Gluc-DH, Merck, Darmstadt, Germany). The incremental area under the insulin curve from the OGTT was calculated with the trapezoidal rule as: Ins_30_ + 2 × Ins_60_ + 2 × Ins_90_ + Ins_120_ – 6 × Ins_0_.

Insulin resistance based on the homeostasis model (HOMA-IR) was computed with the formula: fasting plasma glucose (mmol/L) × fasting serum insulin (mU/L) (14). Insulin-mediated glucose disposal was estimated with a euglycemic insulin clamp as described by DeFronzo (15), with insulin (Actrapid Human, Novo, Copenhagen, Denmark) infused at a constant rate of 56 mU/body surface area (m^2^)/min during 120 minutes. This rate was estimated to suppress hepatic glucose output almost completely also in participants with type 2 diabetes. The target plasma glucose concentration was 5.1 mmol/L. Insulin sensitivity index (M/I) was calculated as glucose disposal rate (mg glucose infused/(min × kg body weight)) divided by the mean plasma insulin concentration (mU/L), during the last 60 min of the 120 min insulin clamp, and multiplied by 100. The unit for M/I is 100 × mg × min^-1^ × kg^-1^/(mU × L^-1^). The OGTT and the euglycemic insulin clamp were performed separated in time by approximately one week (16).

Total plasma 25-hydroxyvitamin D was determined with high-performance liquid chromatography (HPLC) atmospheric pressure chemical ionization mass spectrometry (MS) at Vitas, Oslo, Norway using a HP 1100 liquid chromatograph (Agilent Technologies, Palo Alto CA, USA).

Type 2 diabetes (T2DM) and impaired glucose tolerance (IGT) were defined according to the 1999 World Health Organization (WHO) criteria (16).

### Measurements of temperature

The outdoor temperature (ODT) in degrees Celsius (°C) was recorded at the Swedish Air Force base (F16), located 4 km north of Uppsala center, using calibrated scales. Data were bought from the Swedish Meteorological and Hydrological Institute (SMHI, Norrköping, Sweden) (http://www.smhi.se) as monthly mean values for each month from August 1991 to May 1995. For each participant the mean ODT of the month for the euglycemic insulin clamp investigation and the two preceding months was used as the ODT exposure value.

### Statistical methods

All continuous variables were summarized with number of observations and mean (standard deviation) for winter (October–April) and summer (May–September) seasons.

The reliability of M/I, HOMA-IR, AUC, FPG, 2-h plasma glucose, height, weight, BMI, and WC were displayed as coefficients of variation (CV).

To meet the assumptions of the regression models FPG, 2-h glucose, and BMI were transformed with a logarithmic function, while M/I and AUC were transformed with the square root function.

Values from October 1991 to May 1995 for the continuous variables M/I, HOMA-IR, AUC, FPG and 2-h glucose, energy intake, height, weight, BMI, WC, and vitamin D concentrations were examined for putative seasonal effects with an indicator variable for winter/summer season, in linear regression models. Similarly, smoking, T2DM, IGT, and medication (oral hypoglycemic agents and antihypertensives) were examined for winter/summer effects, in logistic regression models. M/I and HOMA-IR were also examined for the effect of ODT and the combined effect of winter/summer season and ODT. An extended model was estimated where the seasonal effects on M/I and HOMA-IR were adjusted for energy intake, BMI, WC, vitamin D, smoking, T2DM, IGT, and medication. All regression models were adjusted for age at examination. The models were examined for autocorrelation of residuals with the Durbin–Watson test statistic. No adjustment was made when the test statistic was between 1.5 and 2.5 (17).

All statistical tests were two-tailed, and a *P* value of less than 0.05 was considered a statistically significant result. The statistical packages SAS® (v. 9.1) and R (v. 2.8.0) were used.

## Results

### Crude models

During winter, compared with summer season, M/I was 11.0% lower (4.84 versus 5.44 units, *P* = 0.0003), AUC was 16.4% higher (1167 versus 1003 mU/L, *P* = 0.007), WC was 1.4% higher (95.0 versus 93.7 cm, *P* = 0.03), while FPG (5.80 versus 5.71 mmol/L, *P* = 0.28) and 2-h plasma glucose were similar (8.35 versus 8.27 mmol/L, *P* = 0.58). There were no statistically significant differences between winter and summer as regards HOMA-IR, BMI, energy intake, smoking, usual leisure-time PA, glucose tolerance state (T2DM, IGT, or NGT), or medication (oral hypoglycemic agents and antihypertensives) (Table I). All Durbin–Watson test statistic values were between 1.76 and 2.03.

**Table I.** Clinical characteristics of study participants presented for winter and summer seasons and coefficients of variation determined by the reliability study performed.

Variable	Winter season	Summer season	*P*^b^	CV (%)
Temperature (°C)	3.1 (4.4)	10.9 (3.2)	–	
M/I (100 × mg × min^-1^ × kg^-1^/(mU × L^-1^))	4.84 (2.44)	5.44 (2.53)	0.0003	13.9
HOMA-IR (pmol × mmol/L^2^)	77.0 (56.0)	81.0 (72.7)	0.11	15.6
Incremental area under insulin curve (pmol × min/L)	1167 783	1003 (632)	0.007	42.6
Fasting plasma glucose (mmol/L)	5.80 (1.52)	5.71 (1.32)	0.28	5.8
2-h plasma glucose (OGTT) (mmol/L)	8.35 (4.03)	8.27 (4.09)	0.58	14.2
Energy intake (kcal)^a^	1735 456	1772 (467)	0.22	
Height (cm)	174.8 (5.8)	174.7 (6.4)	0.88	0.0
Weight (kg)	80.7 (11.4)	79.6 (11.9)	0.18	0.8
BMI (kg/m^2^)	26.4 (3.4)	26.0 (3.4)	0.12	0.8
Waist circumference (cm)	95.0 (9.5)	93.7 (9.7)	0.03	2.3
25-Hydroxyvitamin D (nmol/L)	66.5 (17.8)	72.5 (20.7)	< 0.0001	
Smoker (%)	20.5	20.9	0.88	
High usual leisure time physical activity (%)	60.0	63.9	0.23	
Type 2 diabetes (%)	14.3	16.0	0.52	
Impaired glucose tolerance (%)	13.1	12.1	0.67	
Diabetes medicine: per oral (%)	5.3	3.4	0.15	
Hypertension medicine: ACE inhibitors (%)	6.1	6.1	0.99	
Hypertension medicine: beta blockers (%)	19.6	17.2	0.33	

Data presented are: Mean (SD) or % for winter season (October–April), *n* = 810; and summer season (May–September), *n* = 307. Coefficients of variation (CV) from the reliability study, *n* = 20.

^a^Data on energy intake is presented from a subset (winter season, *n* = 762; summer season, *n* = 288).

^b^Adjusted for age.

### Adjusted models

M/I was lower (*P* = 0.0006) and AUC was higher (*P* = 0.024) during winter, compared with summer season, with adjustment for age, BMI, WC, energy intake, vitamin D, smoking, glucose tolerance state (T2DM, IGT, or NGT), medication (oral hypoglycemic agents and antihypertensives), and PA.

The AUC, when adjusted for M/I, was 7.2% higher (*P* = 0.30) during winter versus summer season. Thus, variation of insulin sensitivity was compensated for by an insulin secretion variation.

### Effects of outdoor temperature

**Figure 1.**
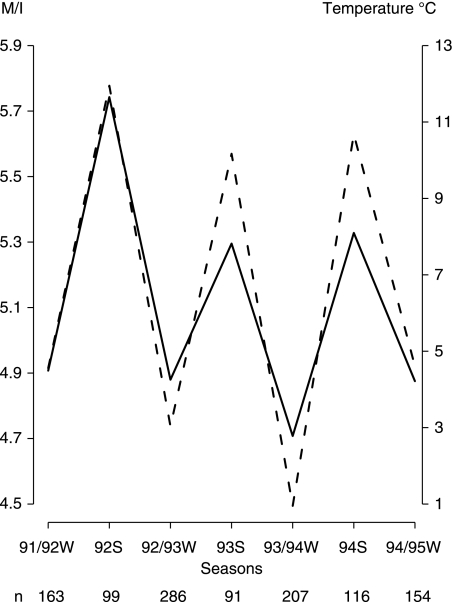
Mean values of insulin sensitivity index M/I (solid line) (100 × mg × min^-1^ × kg^-1^/(mU × L^-1^)) from a euglycemic insulin clamp, and outdoor temperature (dotted line) (degrees Celsius), versus winter (W) and summer (S) seasons of the year from the years 1991 to 1995. Outdoor temperature is the mean of observed outdoor temperatures of the month of the investigation and the 2 months preceding it.

There was an association (*P* < 0.0001) between M/I and ODT (Figure 1). The average increase of M/I was 0.57 units (95% CI 0.29–0.82) per 10°C increase of ODT. The effect of ODT on M/I was statistically significant (*P* = 0.049) when adjusted for winter/summer season, while the effect of the latter did not attain statistical significance (*P* = 0.19). There was no effect of ODT on HOMA-IR.

## Discussion

We found seasonal variations of insulin sensitivity, compensated for by inverse variations of insulin secretion resulting in only small variations of FPG. Insulin sensitivity was lower during the winter season independent of alterations in BMI, WC, energy intake, vitamin D, smoking, glucose tolerance state (T2DM, IGT, or NGT), medication (oral hypoglycemic agents and antihypertensives), and PA; however, the seasonal variations of insulin sensitivity were associated with the outdoor temperature independent of the winter/summer season effects. Previous studies, using surrogate measures of insulin sensitivity, present inconclusive results regarding seasonal variations of insulin sensitivity (4,5,18). Seasonal variation of insulin secretion indicating compensatory elevation during the winter has been observed (19) in a small study of 50-year-old men, using the sum of insulin determinations during an OGTT.

Diabetic patients lack the ability to increase insulin secretion to compensate for a decrease in insulin sensitivity (1–3), and therefore it is likely that the reported increase of HbA1c is largely determined by seasonal variations in insulin sensitivity. This hypothesis should be formally tested in future studies on diabetes patients.

HOMA-IR did not show a seasonal variability. In our reliability study (*n* = 20) the coefficient of variation (CV) for M/I was 13.9%, and 15.6% for HOMA-IR, for which a higher CV of 23.5% is reported (14). Different reliability for M/I and HOMA-IR is a partial explanation of the discrepancy between seasonal effects for these two measures. Further, M/I and HOMA-IR reflect different aspects of insulin sensitivity: M/I primarily skeletal muscle and hepatic to a low extent, whereas HOMA-IR reflects both hepatic and skeletal muscle insulin sensitivity. The temperature effect on insulin sensitivity is present mainly in the peripheral tissue skeletal muscle (20), supported by observed increases of skeletal muscle insulin sensitivity in rats (8) and in diabetic mice (21) by heat treatment.

Basal metabolic rates (7) co-vary with ODT. Antarctic residence, exposing humans to extreme cold, is associated with increased TSH response to TSH-releasing hormone and decreased thyroxin (FT4), which correlate with metabolic markers, such as total and LDL-cholesterol, of thyroid hormone activity in the liver and adipose tissue (22). Low normal FT4 levels are also associated with decreased insulin sensitivity (23). These hormonal alterations may contribute to the greater impact of ODT than winter/summer season on insulin sensitivity fluctuations. Increase in active vitamin D induced by sunlight exposure during the summer is directly associated with insulin sensitivity (24), but adjustment for vitamin D did not alter the winter/summer difference of insulin sensitivity. Vitamin D was determined with HPLC method, which, in a study by Snellman et al. (25), proves to have very good reliability. Energy intake was measured with a validated optically readable 7-day food record (12). Dietary intake is difficult to measure with high precision, but, except for methods based on food weighing, our method is the most accurate. Thus, lack of precision and resulting residual confounding probably cannot explain that adjustments for vitamin D and energy intake did not remove the seasonal effects on insulin sensitivity.

Alterations in physical activity are closely related to ODT and winter/summer seasons and also a strong determinant of insulin sensitivity. Increased insulin sensitivity during summer may be due to more intense physical activity. However, the participants provided data only on average yearly estimates of leisure time physical activity. As gardening during summer and snow shoveling during winter were physical activities frequently performed by study participants, in addition to walking, one may consider that physical activity in this age-group of retired men would be less variable over the calendar year than in younger people.

The strength of these results is that the ULSAM study is large and population-based, investigated with the euglycemic insulin clamp to assess insulin sensitivity. Investigations were performed around the calendar year for 4 years. The population represents a homogeneous sample for age, gender, and ethnicity. Weaknesses are that extrapolation to women, younger individuals, and other ethnic groups must be made with caution and that we lack data on TSH.

In conclusion, seasonal variations of insulin sensitivity, measured with euglycemic insulin clamp, were compensated for by inverse variations of insulin secretion resulting in only small variations of plasma glucose. These phenomena should be further investigated in diabetic patients.
